# Fungal infection profile in critically ill COVID-19 patients: a prospective study at a large teaching hospital in a middle-income country

**DOI:** 10.1186/s12879-023-08226-8

**Published:** 2023-04-18

**Authors:** Essamedin M. Negm, Mohamed Sorour Mohamed, Rehab A. Rabie, Walaa S. Fouad, Ahmed Beniamen, Ahmed Mosallem, Ahmed E. Tawfik, Hussein M. Salama

**Affiliations:** 1 Anaesthesia, Intensive Care, and Pain Management Department, Faculty of Medicine, Zagazig University Hospital, Zagazig, Egypt; 2 Internal Medicine Department, Faculty of Medicine, Zagazig University Hospital, Zagazig, Egypt; 3 Medical Microbiology and Immunology Department, Faculty of Medicine, Zagazig University Hospital, Zagazig, Egypt; 4 Family Medicine Department, Faculty of Medicine, Zagazig University Hospital, Zagazig, Egypt; 5 Zagazig University Hospital, Zagazig, Egypt

**Keywords:** COVID-19, ICU, Fungal coinfection, Incidence, Risk factors, Outcome

## Abstract

**Background:**

Critically ill COVID-19 patients are highly susceptible to opportunistic fungal infection due to many factors, including virus-induced immune dysregulation, host-related comorbidities, overuse and misuse of antibiotics or corticosteroids, immune modulator drugs, and the emergencies caused by the pandemic. This study aimed to assess the incidence, identify the potential risk factors, and examine the impact of fungal coinfection on the outcomes of COVID-19 patients admitted to the intensive care unit (ICU).

**Methods:**

A prospective cohort study including 253 critically ill COVID-19 patients aged 18 years or older admitted to the isolation ICU of Zagazig University Hospitals over a 4-month period from May 2021 to August 2021 was conducted. The detection of a fungal infection was carried out.

**Results:**

Eighty-three (83) patients (32.8%) were diagnosed with a fungal coinfection. Candida was the most frequently isolated fungus in 61 (24.1%) of 253 critically ill COVID-19 patients, followed by molds, which included Aspergillus 11 (4.3%) and mucormycosis in five patients (1.97%), and six patients (2.4%) diagnosed with other rare fungi. Poor diabetic control, prolonged or high-dose steroids, and multiple comorbidities were all possible risk factors for fungal coinfection [OR (95% CI) = 10.21 (3.43–30.39), 14.1 (5.67–35.10), 14.57 (5.83–33.78), and 4.57 (1.83–14.88), respectively].

**Conclusion:**

Fungal coinfection is a common complication of critically ill COVID-19 patients admitted to the ICU. Candidiasis, aspergillosis, and mucormycosis are the most common COVID-19-associated fungal infections and have a great impact on mortality rates.

## Introduction

For healthcare systems, particularly intensive care units (ICU) throughout the world, the Corona Virus disease 2019 (COVID-19) pandemic poses an unprecedented challenge and a tremendous load. A higher risk of serious illness and mortality has been linked to comorbidities, malignancies, organ transplantation, and other immunocompromising conditions. C-reactive protein (CRP), ferritin, D-dimer, interleukin-6 (IL-6), oxygenation (PaO2 and SaO2), and many other clinical and laboratory scores have been used to predict the outcome of COVID-19 [1–3].

Due to its resemblance to other viral respiratory processes, bacterial and fungal coinfections and superinfections range widely and have the potential to signal the progression of COVID-19. In the SARS-CoV-1 virus pandemic, bacterial coinfection was 22%; in influenza virus pandemics, bacterial coinfection varied from 2 to 65%; and in fungal coinfection, it ranged from 15 to 25% [4–6].

Admitted patients with COVID-19 are highly susceptible to fungal infection. First, due to infection of enterocytes and alteration of intestinal hemostasis with tissue destruction; second, the immune system’s dysregulation and the high release of cytokines, Third, by associated comorbidities such as chronic obstructive pulmonary disease (COPD), diabetes, chronic kidney disease (CKD), immunosuppressive therapy, and the usage of invasive medical devices [7, 8].

SARS-CoV-2 targets angiotensin-converting enzyme 2 (ACE2), and transmembrane protease serine 2 (TMPRSS2)-expressing cells cause airway epithelial damage with the expression of apical receptors such as integrins. This, in turn, mediates interactions with proteins on the surface of the Mucorales, Aspergillus, or Candida cell wall, namely the spore-coating (CotH) proteins, the thaumatin-like protein CalA, and mannoproteins, to enhance fungal adhesion and invasion. The activation of antiviral immunity following viral recognition by innate immunity may also paradoxically promote systemic inflammatory responses and create a highly permissive environment for developing fungal co-infections. The severe lymphopenia and lymphocyte dysfunction commonly seen in COVID-19 may also impact the growth of fungal co-infections [9]. However, additional factors contribute to the high prevalence of fungal infection in COVID-19 patients, such as pandemic-related emergencies, overcrowding in the medical system, a lack of trained staff, and work stress, which make it challenging to implement pre-pandemic infection control measures and encourage the emergence of nosocomial outbreaks [10].

The reported incidence of fungal infection in COVID-19 patients is 3.7%, while cases admitted to the ICU had a higher incidence (9.6%) with increased fatalities [11, 12]. General awareness of the significance of fungal co-infection is crucial to prevent delays in diagnosis and treatment, which will help minimise morbidity and death from opportunistic invasive fungal infections (IFIs). Antifungal medications are now accessible, but their usage is challenging due to their cost, route of administration, and toxicity, which is problematic for low- and middle-income nations in particular [9, 13].

So, the objectives of this study were: to know the incidence of fungal coinfection/superinfection among patients with COVID-19 admitted to the Zagazig University Hospitals (ZUH) isolation ICUs, and to identify predictors of infection and rates of mortality.

## Patients and methods

### Study design

This prospective cohort study was conducted in the isolation ICU of ZUHs, Egypt, over four months during the third wave of COVID-19, from May 2021 to August 2021. The East Delta, Suez Canal, and Sinai governorates are served by the tertiary care teaching hospitals of ZU, with a bed capacity of 2,250 beds. There were 27 ICU beds available when the data were collected. This study was approved by the Institutional Review Board (IRB), Faculty of Medicine, ZU (IRB reference number: ZU-IRB#: 6847-30-3-2021). We followed the ethical principles of the Declaration of Helsinki during the preparation for this study. Informed consent was obtained from all study participants or their legal guardians.

### Patient enrollment

The current study included 253 critical COVID-19 patients aged 18 years or older admitted to the ICU and confirmed by the real-time PCR on the nasopharyngeal swab specimens. All participants’ detailed histories were recorded, including data on age, sex, body mass index (BMI), residence, and clinical examination data focusing on severity, oxygen saturation, and associated risk factors as underlying comorbidities. All patients were investigated by total leucocytic count, ferritin level, d-dimer, and serum inflammatory markers, including CRP. Patients’ medications and length of hospital stay were also recorded. The management of COVID-19 patients and sepsis was carried out in accordance with local and international guidelines and protocols [14, 15]. Methylprednisolone was the steroid used following our protocol, at a dosage of 1–2 mg/kg/day; 1 mg/kg/day for non-ventilated patients and 2 mg/kg/day for ventilated patients. Tocilizumab was administered in doses of 4–8 mg/kg/day, spaced 12–24 h apart. The high-dose corticosteroids were methylprednisolone > 100 mg/day or an equivalent [16].

A local policy for fungal infection management was established following international standards and recommendations [17–19]. Bacterial infections are managed in accordance with the current local antimicrobial policy, which was established based on the 2021 local antibiogram [20].

The treating physician’s clinical judgement was used to establish the location of the suspected fungal site infection for targeted specimen collection, the start, de-escalation, and stoppage of antifungal treatment. Additionally, any adjustments to the dosage of steroids or antibiotics were related to the treating physicians.

#### Detection of SARS-CoV2 using reverse transcription real-time polymerase chain reaction (RT-PCR)

A nasopharyngeal and an oropharyngeal swab were obtained from every patient under complete aseptic conditions. Then placed in 3 mL of the viral transport medium (VTM, Ismailia free zone, Egypt) and immediately sent to the Scientific and Medical Research Centre, Faculty of Medicine, Zagazig University. Viral RNA was extracted using a viral RNA mini kit (Qiagen, Japan, cat. no. 52,906) according to the manufacturer’s instructions. Then, the RNA-dependent RNA polymerase gene of SARS-CoV2 was targeted with a one-step RT-qPCR using a real-time PCR kit (Primerdesign Ltd., UK) in a Stratagene Mx3000P qPCR System (Agilent, USA). A total volume of 20 µL mixture of 2X RT-qPCR Master Mix (10 µL), Primer & Probe (2 µL), and RNA extract (8 µL). Positive and negative controls were included in each run. The reverse transcription was carried out by heating the samples at 55 ◦C for 10 min. Following initial denaturation (heating at 95 ◦C for 2 min), 45 cycles of denaturation (at 95 ◦C for 10 s), annealing, and extension (at 60 ◦C for 1 min) were performed. The sample cycle threshold (Ct) value was recorded. If it was ≥ 40 or not recorded, then the sample was considered negative [21].

#### Detection of fungal superinfection

Early morning sputum, endotracheal aspirate (ETA), nasopharyngeal swabs, oropharyngeal swabs, urine, and blood for blood culture were collected in appropriate containers according to the standard protocol with strict adherence to the recommendations of the Centers for Disease Control and Prevention (CDC) during collection, transportation, and processing of specimens [22]. Specimens were rapidly transferred to the microbiological laboratory.

Specimens were subjected to microscopic examination using 10% KOH as well as Gram stain for screening of fungal elements (hyphae, pseudohyphae, and yeast cells). Then cultured, in duplicate culture plates, on Sabouraud dextrose agar (SDA) with chloramphenicol without cycloheximide (Oxoid, Basingstoke, UK), each incubated at 37 ◦C and room temperature in addition to blood agar at 37◦C. Blood cultures were incubated at 37 ◦C and positive cultures were inoculated on blood agar and SDA plates.

*Candida* was suspected by round to oval cells, and sometimes pseudohyphae were detected on the Gram-stained film. Also, culture clarified the presence of *Candida* colonies. Then further investigation was done by germ tube test, and CHROMagar Candida (CHROMagar, France) was used to differentiate candida spp.

Filamentous fungi were identified according to their macroscopic and microscopic criteria using lactophenol cotton blue (LPCB). *Aspergillus Fumigatus* was identified by its rapid growth with a velvety or powdery texture, at first white, then turning green to gray, with a pale-yellow reverse. Microscopically, hyaline septate unbranched hyphae with short smooth conidiophores terminate in a dome-shaped vesicle. Phialides are flask-shaped uniseriate on the upper two-thirds of the vesicle. *Aspergillus Flavus* was characterized by a velvety texture that ranged from yellow to green or brown with a goldish to brown reverse. Microscopically, a rough and spiny conidiophore with uniseriate or biseriate phialides covers the entire vesicle and points out in all directions.

*Mucor* was characterized by its rapid growth of white, fluffy colonies that turned grey to brown with age and had a pale or yellow reverse. Microscopically, it showed broad, aseptate hyphae branching at wide angles (> 90◦) with terminal, round, spore-filled sporangia, supported by columellae of variable shapes and light to pigmented in colors with no rhizoids.

*Alternaria* was identified microscopically by its dark hyphae, chained poroconidia, horizontal and vertical septa, and club-shaped base with tapered apices. Macroscopically, the light grey, woolly colony rapidly changes to dark greenish black with a black reverse.

*Penicillium* is characterized by rapid-growing colonies that are initially velvety and white, later becoming powdery blue-green with a white periphery and colorless reverse. Microscopically, hyaline septate hyphae, with conidiophore bearing up to five metulae, the wide phialides born in groups of four to six on the metulae, which in turn support lemon-shaped phialoconidia in chains.

*Fusarium* was identified by the rapidly growing colony, which was white at first and woolly or cottony later, becoming lavender, yellow, or orange with a light reverse. Microscopically, hyaline septate hyphae, diagnostic macrophialoconidia are two- to five-celled banana-shaped, with a foot cell at the point of attachment.

#### Definition of probable invasive aspergillosis in the intensive care unit

We Followed the EORTC/MSGERC approach for diagnosis of the probable invasive aspergillosis for ICU patients [23].The diagnosis based on mycological evidence of *Aspergillus* spp. in a lower respiratory tract specimen by direct microscopy, and culture, beside the clinical and radiological abnormalities.

#### Statistical analysis

All results were analyzed using IBM SPSS 23.0 (SPSS Inc., Chicago, USA). Continuous data were presented as mean ± standard deviation (SD) whereas categorical data were presented as frequency and percentage. The chi-square test (X^2^) and fisher exact test were used to compare proportions. Odds ratios (ORs) with 95% confidence intervals (95% CI) were calculated for each significant variable based on multivariate logistic regression [24]. Significance was indicated by a p-value ≤ 0.05.

## Results

This study enrolled 253 confirmed COVID-19 patients; admitted to the isolation ICU of ZU hospitals; 146 males (57.7%) and 107 females (42.3%), with ages ranging from 20 to 84 years. All the cases were from the Sharkia governorate. Of all participants, 22.1% were smokers. About half of the patients (46.6%) have underlying medical problems; hypertension is the most frequently associated comorbidity (42.7%), followed by diabetes mellitus in more than one-third of patients (38.3%), ischemic heart diseases (10.3%), chronic hepatic disease (6.7%), chronic kidney disease (9.9%), underlying malignancy (5.1%), COPD (7.1%), and solid organ transplantation (1.3%). More than one-third (37.5%) of patients have multiple comorbidities (more than two). About 26.8% of patients used steroids before ICU admission (chronic or recent usage). A high dose of steroids was used in 103 patients (40.7%). Tocilizumab was used in the treatment protocol in 63 patients (24.9%) (Table 1).

**Table 1:** Demographic characteristics and underlying conditions of the studied participants

Variable	Studied participants (n = 253)	
Age (years):		
Mean ± SD (Range)	53.76 ± 11.43 (20–84)	
Sex:	**n**	**%**
Male Female	146 107	(57.7) (42.3)
Smoking	56	(22.1)
Associated comorbidities	118	(46.6)
Diabetes mellitus	97	(38.3)
Hypertension	108	(42.7)
Ischemic heart disease	26	(10.3)
Chronic liver disease	17	(6.7)
Chronic kidney diseases	25	(9.9)
Malignancy	13	( 5.1)
Chronic obstructive pulmonary disease	18	( 7.1)
Organ transplantation	3	( 1.3)
Multiple comorbidities ≥ 3	95	(37.5)
Steroid usage before ICU admission	68	(26.8)
Use of high-dose steroids	103	(40.7)
Use of Tocilizumab	63	(24.9)

Among 253 critically ill COVID-19 patients admitted to the ICU, 167 (66%) had oropharyngeal candidiasis (OPC), but 83 (32.8%) had other sites fungal coinfection, including two patients with clinically and radiologically diagnosed mucormycosis (rhino and occulo cerebral).Candida was isolated in 61 (24.1%) of the patients, followed by molds, including Aspergillus 11 (4.3%) and mucormycosis in five (1.97%), and six (2.4%) patients were diagnosed with other rare fungi. Candida represents 73.5% (61/83) of fungal infection cases and most of the Candida isolates were Candida albicans (70.5%). Localized noninvasive oropharyngeal candidiasis represented 41.9% (106 patients) of total cases and was found to be associated with all fungal infections of other sites. (Table 2).

**Table 2:** Types of fungal pathogens in patients with COVID 19

Oropharyngeal *Candidiasis* (n = 167/253)	Localized Oropharyngeal *Candidiasis* (n = 106/167) Oropharyngeal with other sites Candidiasis (n = 61/167) Oropharyngeal *Candidiasis* with other sites of fungal infection (n = 83/167)	N	%
		167	66%
Other sites of fungal infection (n = 83/253) (32.8%)			
	*Candida* species (61/83) Candida albicans (43/61) Candida non albicans (18/61) Candida krusei (10/61) Candida parapsilosis (7/65) Candida tropicalis (1/65)	*61*	24.1%
	Aspergillus species (11/83) Aspergillosis Fumigatus (9/11) Aspergillosis Flavus (2/11)	11	4.3%
	*Mucormycosis* (5/83)	*5*	1.97%
	Other fungal infection (6/83) Alternaria species (3/83) Fusarium species (2/83) Penicillium species (1/83)	6	2.4%

Most candida infections were localized in the lung (20/61) and urinary tract (17/61), and six cases had both pulmonary and urinary candidiasis; 18/61 cases of candida infection invaded the blood from the pulmonary or urinary tract and caused candidemia; all aspergillosis and three cases of mucormycosis were pulmonary, and the other two cases were oculocerebral and rhinocerebral mucormycosis (Table 3).

**Table 3:** Distribution and time of diagnosis of fungal infection in the studied patients

Type of fungal infection	Distribution of fungal infection							Days between ICU admission and fungal infection diagnosis (mean ± SD)
	Sputum		Urine	Blood	Sputum + urine	Urine + Blood	Sputum + Blood	
*Candidiasis* (n = 61)	Candidiasis of isolated other sites (n = 43)				Candidiasis of multiple other sites (n = 18)			9.96 ± 4.92
	(20)	(17)		(6)	(6)	(5)	(7)	
Aspergillosis (n = 11)	(11)	(0)		(0)	(0)	(0)	(0)	18.36 ± 5.77
*Mucormycosis* (n = 5)	(3)	(0)		(0)	(0)	(0)	(0)	22.6 ± 2.51
		Other sites						
		(1) Oculo-Cerebral				(1) Rhino- cerebral		
Other fungal infections (n = 6)	(3) Alternaria (2) Fusarium (1) Penicillium							13.33 ± 8.50 17.5 ± 7.77 23 ± 0

When the relationship between the risk factors and the occurrence of fungal infections was investigated, there was no significant difference between COVID-19 patients and those without fungal coinfection regarding age, sex, smoking habits, and body mass index. However, we detected a highly statistically significant association with fungal infection among patients with chronic liver disease, patients with multiple comorbidities (p-value ≤ 0.001), organ transplantation (p = 0.001), malignancy (p = 0.005), and COPD (p = 0.04). However, no significant difference was found regarding other comorbidities, including hypertension, ischemic heart disease, and chronic kidney disease. Diabetes mellitus was not associated with fungal coinfection (p = 0.18), but it was associated with poor diabetic control (p-value 0.001).COVID-19 patients with fungal coinfection have higher baseline APACHE II and SOFA scores than COVID-19 patients without fungal coinfection (p-values of 0.001 and 0.01 respectively), but no significant differences in the P/F ratio at ICU admission (p-value of 0.8) (Table 4).

**Table 4:** Baseline clinical and laboratory risk parameters in the studied patients:

Risk parameters	fungal infections		P value
	**Positive** (n = 83)	**Negative** (n = 170)	
	**n (%)**	**n (%)**	
Age **(yr)** (mean ± SD)	(55.16 ± 11.73)	(54.9 ± 10.28)	0.76
Sex			
Male (n = 146) Female (n = 107)	52 (62.7) 31 (37.3)	93 (54.7) 77 (45.3)	0.18
Smoking (n = 56)	20 (24.1)	36(21.2)	0.65
Diabetes mellitus (n = 97)	39(46.98)	58 (34.1)	0. 08
Poorly controlled DM (n = 36)	29 (34.9)	7 (4.1)	0.001*
Hypertension (n = 108)	36 (43.4)	72(42.4)	0.71
Ischemic heart disease (n = 26)	9(10.8)	17(10)	0.86
Chronic kidney diseases (n = 25)	10 (12.05)	15(8.8)	0.16
Malignancy (n = 13)	10 (12.05)	3 (1.8)	0.005*
Chronic obstructive pulmonary disease (n = 18)	9(10.8)	9(5.3)	0.04 *
Chronic liver disease (n = 17)	9 (10.8)	8(4.7)	0.03*
Organ transplantation (n = 3)	3 (3.6)	0 (0)	0.001*
Multiple comorbidities ≥ 3 (n = 67)	51 (61.4)	16(9.4)	0.001*
APACHE II Score (mean ± SD)	15.3 ± 5.4	7.5 ± 3.1	0.001*
SOFA Score (mean ± SD)	6.4 ± 3.2	3.4 ± 2.4	0.01*
Body mass index (kg/m2) (mean ± SD)	29.6 ± 3.45	24.5 ± 2.65	0.12
PaO2/FiO2 Ratio (P/F ratio)	116 ± 105.5	125 ± 118.5	0.8
Laboratory findings at COVID-19 diagnosis (mean ± SD) :			
white blood cell count	9.14 ± 2.4	11 ± 3.1	0.13
Lymphocytic %	11 ± 7.41	13.08 ± 8.46	0.17
CRP (mg/l)	96 ± 45.7	24 ± 14.08	0.01*
D-dimer (ug/mL)	2.3 ± 1.01	1.1 ± 1.4	0.05*
Ferritin (ng/ml)	684.73 ± 469.22	298.61 ± 186.05	0.01*
Albumin (g/dL)	3.1 ± 0.61	3.4 ± 052)	0.14

Statistically significant (*) (p ≤ 0.05): high statistical significant (p ≤ 0.01)

DM : Diabetes mellitus ,APACHE: acute physiology and chronic health evaluation, SOFA: sequential organ failure assessment,, CRP: c- reactive protein

As regards the baseline laboratory data; there was a statistically significant difference between both groups in CRP, D-dimer, and serum ferritin (p-values of 0.01; 0.05; and 0.01 respectively), with the higher values in the fungal positive group, but no significant differences were found in the CBC or serum albumin level (p-values of 0.13, 0.7, and 0.14 respectively) (Table 4).

Regarding drug therapy as a risk factor for fungal coinfection; all of the study patients were critically ill and received steroids and at least one antibiotic, there was a significant association between fungal coinfection and prolonged steroid use for more than three weeks, use of a high-dose steroid, and the use of tocilizumab (p ≤ 0.001). There was a significant association between fungal coinfection and the use of antibiotic combinations and the longer duration of antibiotic therapy (p-values of 0.03 and 0.02, respectively). Other significant risk factors for fungal coinfection included invasive and noninvasive mechanical ventilation (p-values of 0.04 and 0.001, respectively); central venous line (p-value ≤ 0.001); parenteral nutrition (p-value = 0.008); and the length of hospital stay (p-value = 0.001) (Table 5).

**Table 5:** Medications and invasive procedures used in treatment of the studied patients

	fungal infection		P value
	**Positive** (n = 83)	**Negative** (n = 170)	
	**n (%)**	**n (%)**	
**Medications**			
High dose steroid (n = 113)	63(75.9)	50 (29.9)	< 0.001*
Usual steroid dose	20 (24.1)	120 (70.6)	< 0.001*
Prolonged steroid use (> 3 week) (n = 42)	27 (32.5)	15 (8.8)	< 0.001*
Tocilizumab (n = 63)	41(49.4)	22(12.9)	< 0.001*
Antibiotic use	83 (100)	170(100)	
Antibiotic combination	61(73.5)	74 (43.5)	0.01*
Duration of antibiotic use	11 ± 7.4	7 ± 2.7	0.02*
**Invasive devices**			
Noninvasive MV (n = 135)	68(81.9)	67(39.4)	0.04*
Invasive MV (n = 85)	62(74.7)	23 (13.5)	< 0.001*
Central venous line	57 (68.8)	31(18.2)	0.001*
Parenteral nutrition	47 (56.6)	31(18.2)	0.008*
Length of hospital stay: median (range)	13.5 (2–47)	6.9 (2–24)	< 0.001*

Statistically significant (*) (p ≤ 0.05): high statistical significant (p ≤ 0.01)

MV: mechanical ventilation

Multivariate analysis was performed to detect possible risk factors associated with fungal superinfection among the studied participants. Poor diabetes control, prolonged and/or high-dose steroids had high statistical significance (p-value = 0.001), as did the presence of multiple comorbidities (p-value = 0.004) (Table 6).

**Table 6:** multivariate logistic regression to detect risk factors for fungal infections

	Beta	S. E	Wald	P	Odds ratio	95% C.I.for EXP(B)		
						Lower		Upper
Poor diabetic control	2.323	0.557	17.415	0.001*	10.207	3.428	30.392	
Malignancy	1.183	0.671	3.104	0.078	3.264	0.875	12.171	
Chronic obstructive pulmonary disease	-1.803	0.960	3.526	0.060	0.165	0.025	0.982	
Chronic liver disease	0.881	0.992	0.788	0.375	2.413	0.345	16.858	
Organ transplantation	0.617	0.881	0.490	0.484	1.853	0.330	10.412	
Multiple comorbidities	2.115	0.846	12.657	0.004*	4.574	1.834	14.883	
APACHE II Score	0.699	1.031	0.459	0.498	2.011	0.266	15.183	
SOFA Score	0.283	0.670	0.178	0.673	1.327	0.357	4.937	
Prolonged steroid use	2.646	0.465	32.347	0.001*	14.1	5.665	35.097	
Use of high dose steroids	2.648	0.856	16.657	0.001*	14.574	5.834	33.784	
Use of tocilizumab	0.701	0.811	0.645	0.42	2.015	0.365	11.142	
Antibiotic combination	1.610	1.184	1.850	0.174	5.004	0.492	50.947	
Noninvasive MV	1.758	0.874	4.784	0.06	6.351	1.634	15.875	
Invasive MV	0.287	0.641	0.200	0.654	1.332	0.380	4.676	
Central venous line	1.136	0.752	2.283	0.051	3.115	0.714	13.598	
Parenteral nutrition	1.798	0.934	5.729	0.093	4.221	1.365	11.142	
Duration of antibiotic use	0.701	0.872	0.645	0.422	2.015	0.365	11.142	
Length of hospital stay	2.401	1.257	3.648	0.056	11.032	0.939	12.953	
CRP	0.633	0.601	1.109	0.292	1.883	0.580	6.117	
D-dimer	1.136	0.752	2.283	0.131	3.115	0.714	13.598	
Ferritin	0.118	0.699	0.028	0.866	1.125	0.286	4.430	

Statistically significant (*) (p ≤ 0.05): high statistical significant (p ≤ 0.01)

C.I: confidence interval,O.R: odds ratio,S.E: standard error, APACHE: acute physiology and chronic health evaluation, SOFA: sequential organ failure assessment, MV: mechanical ventilation, CRP: c- reactive protein

As regards the outcomes of the studied patients, 75 (29.6%) of the total number of participants died, including 53/83 (63.9%) patients with fungal infections and 22/170 (12.9%) patients without an associated fungal coinfection (Table 7). The mortality rates varied according to the type of fungal coinfection: 7/20 (35%) of patients with pulmonary and 6/17 (35.3%) of patients with urinary *candidiasis* died. The mortality rate was markedly increased in the patients with *candidemia* and multiple-site *candidiasis*, including those with blood, sputum, and/or urine (83.3% and 88.9%, respectively). **Table 7** shows that 81.8% of Aspergillosis patients and 100% of mucormycosis patients died.

Patients with fungal coinfection, particularly mucormycosis, have a longer hospital stay than negative patients; the indication of both invasive and non-invasive mechanical ventilation was higher in fungal infection patients (Tables 5 and 7).

**Table 7:** Effect of fungal infection on patients’ outcomes

Total case (n = 253)	Negative Fungal (n = 170)	Positive Fungal (n = 83)	*Candidia*sis (n = 61)				*Aspergillosis* (n = 11)	*Mucormycosis* (n = 5)	Other fungi (n = 6)
			*Blood*	Sputum	Urine	Multisite infection			
Noninvasive MV (n = 135)	67/170 (39.4%)	68/83 (81.9%)	5/6 (83.3%)	15/20 (75%)	12/17 (70.6%)	17/18 (94.4%)	9/11 (81.8%)	5/5 (100%)	5/6 (83.3%)
Invasive MV (n = 79)	24/170 (14.1%)	55/83 (66.2%)	5/6 (83.3%)	8/20 (40%)	7/17 (41.2%)	16/18 (88.9%)	9/11 (81.8%)	5/5 (100%)	5/6 (83.3%)
Length of hospital stay: median (range)	6.9 (2–24)	13.5 (2–47)	11.9 (7–25)	14.1 (2–47)	15.5 (4–21)	13.4 (3–25)	13.6 (7–20)	17.6 (14–20)	12.7 (2–19)
Case fatality (n = 75/253) (29.6%)	22/170 (12.9%)	53/83 (63.9%)	5/6 (83.3%)	7/20 35%	6/17 (35.3%)	16/18 (88.9%)	9/11 (81.8%)	5/5 (100%)	5/6 (83.3%)

## Discussion

The greatest challenge in the history of intensive care medicine has been treating COVID-19 pneumonia in critically ill patients [25]. Patients with COVID-19 have a wide range of symptoms, from asymptomatic disease or mild symptoms to critically ill patients with acute respiratory distress syndrome (ARDS) necessitating ICU admission [26].

Critically ill COVID-19 patients who are admitted to the ICU are highly vulnerable to developing secondary bacterial and fungal infections, which may contribute significantly to an unfavourable prognosis [27]. Severe COVID-9 infections, even in the absence of other factors, can suppress the immune system by altering the T-cell response in a variety of ways [28].

The increased susceptibility of critically ill COVID-19 patients to opportunistic fungal infection is related to numerous factors, including the extensive and irrational use of antibiotics and corticosteroids, associated comorbidities, and invasive medical devices such as central venous catheters, total parenteral nutrition, and invasive and noninvasive mechanical ventilation. The skin barrier is penetrated by these medical devices, providing direct access to the host’s interior [29, 30]. In high-risk patients or those who are not responding to standard therapies, to avoid ignoring fungal infections, clinicians should recognize them as a differential diagnosis and obtain confirmatory laboratory testing [13].

In this study, we assessed the occurrence of fungal coinfections or superinfections in patients with severe COVID-19 admitted to the ICU and investigated the possible contributing risk factors for infection and the impact of fungal infection on the outcome.

Zagazig University ICU’s fungal growth was less than 1% before the COVID-19 era, as recorded in its antibiogram conducted in 2019 [20].

A quite large proportion (66%) of patients got OPC in the current report. General risk factors such as old age, immune dysregulation, prolonged hospitalization, mechanical ventilation, poor oral hygiene, corticosteroid use, overuse of broad-spectrum antibiotics uncontrolled diabetes, malignancy, hypertension, antihypertensive drugs causing xerostomia, chloroquine intake, lymphopaenia, and a lack of mouth brushing for cleaning the teeth, buccal cavity, dentures, and tongue may all predispose COVID-19 patients to develop OPC. To avoid OPC, dentures should be washed each day and left for at least 6 h overnight. Another potential cause of the high prevalence of OPC is dehydration caused by fever and a dry mouth due to the high respiratory rate [30, 31].

In the present study, the overall rate of non-localized fungal coinfection in critically ill COVID-19 patients admitted to the ICU (32.8%) was much higher compared to rates reported in many other types of literature.

Many studies reported high rates of fungal coinfection ranging from 10 to 29% [10, 32]. Data from Hungary showed that 22.2% of all critically ill adult COVID-19 patients hospitalized in a single ICU were diagnosed with IFIs [33]. White et al. [12] reported a higher prevalence of 26.7% IFIs in critically ill COVID-19 patients.

In contrast, Elsawy et al. [34] reported a much lower incidence of fungal respiratory coinfection (4.7%) in Saudi Arabia, but all admitted patients (ICU and non-ICU) participated in the study; similar results from France showed the incidence of fungal respiratory complications (4.8%) of ICU-admitted COVID-19 patients [35]; in China, Zhang et al. [36] reported a fungal coinfection rate of 3.2%.

Understanding candidiasis’ relationship with an inclination in COVID-19 patients necessitates knowledge of its pathogenesis. Inflammation causes an imbalance in iron homeostasis in COVID-19 patients. A reduction in the level of circulating iron and high ferritin levels characterize this disorder. Low iron levels are frequently associated with oral candidiasis. Hyperferritinemia has been linked to ferroptosis and organ damage as a result of TNF-antagonist use, making COVID-19 patients more susceptible to fungal coinfections [37].

In this study, Candida was found to be the most common fungal isolate, accounting for 61/153 (24.1%) of all cases, followed by Aspergillosis (11/253 (4.3%)) and mucormycosis (5/253 (1.97%).More than half of the *Candida* isolates were *Candida albicans* 43/61 (70.5%), then *C. krusei* 10/61 (16.4%), followed by *C. parapsilosis* 7/61 (11.5%). Finally, C. tropicalis accounted for 1/61 (1.6%) of the total. This agrees with results by Khalil et al. [30], who reported *Candida albicans* was the most common isolated species, accounting for 74.36%, followed by *Candida tropicalis* and *Candida glabrata* (15.38% and 10.26%, respectively) in Egyptian patients. Also, results from Saudi Arabia concluded that *Candida albicans* is the most prevalent fungal isolate in about 68.4% of cases of fungal infection, followed by *Candida non-albicans* in 21.1% of cases, and then aspergillosis in 3.5% of cases [34]. Also, Salehi et al. [38] found *that Candida albican*s was the most common pathogen, accounting for 70.7% of cases of fungal coinfection. But, Tokak et al. [39] detected that candidemia is caused by *Candida parapsilosis* (48.4%), followed by *Candida albicans* (32.3%). Similar results from Turkey showed that *Candida parapsilosis* was the leading agent of candidemia (43%), followed by *Candida tropicalis* (31%), and *Candida albican* (25%) [40].

According to a recent consensus recommendation from the European Confederation of Medical Mycology and the International Society for Human and Animal Mycology, severe SARS-CoV-2 infection damages the airways’ lining, allowing Aspergillus fungus to infiltrate the tissues. International research that was published in the CDC journal Emerging Infectious Diseases found that 15% of COVID-19 patients who are admitted to the ICU get an Aspergillus infection. Also, Aspergillus’ resistance to voriconazole and isavuconazole, the first-line treatments, has prompted concern due to the potential for worsening patient outcomes from these secondary fungal infections [41].

According to the results of this study, Aspergillus was the second cause of fungal coinfection (4.3%), followed by mucormycosis (1.97%). Similar results from Wales reported an incidence of 12.6% for aspergillosis among critically ill COVID-19 patients [12]. In Italy, Bartoletti et al. [42] reported a higher incidence of pulmonary aspergillosis (27.7%) among COVID-19 patients requiring invasive mechanical ventilation. Ezeokol et al. [43] concluded that fungal coinfections are reported in severely ill COVID-19 patients admitted to the ICU, with a higher rate of incidence for aspergillosis followed by candidemia. In contrast, a systematic review from Iran showed that the most common fungal infections reported in Iranian COVID-19 patients were candidiasis, followed by mucormycosis and aspergillosis [13].

The results of the current study showed a statistically significant association between fungal infection and multiple comorbidities, chronic liver disease, malignancy, organ transplantation, COPD, and patients with poor diabetic control; also, patients with higher baseline APACHE II and SOFA scores were more susceptible to fungal infection; however, no significant difference was found regarding age, gender, smoking habits, or other comorbidities, including hypertension; ischemic heart disease; body mass index; chronic kidney disease, and well-controlled DM. These findings are consistent with numerous studies that have concluded that critically ill COVID-19 patients typically have more comorbidities that may predispose them to fungal infections [43–45]. Structural lung diseases might predispose them to the development of COVID-19-associated pulmonary aspergillosis (CAPA) [45, 46], as some studies reported a higher incidence in patients with COPD or asthma [47]. Poorly controlled diabetes and diabetic ketoacidosis have been linked to an increased risk of mucormycosis in COVID-19 patients [48].Free,unbound iron in the serum also contributes to mucormycosis development in addition to uncontrolled hyperglycemia. Higher levels of serum-free iron have been linked to acidosis and proton-mediated displacement of ferric iron from transfer in diabetic ketoacidosis, both of which are major risk factors for mucormycosis [49].Also, Moorthy et al. [50] concluded that there is a significant increase in the incidence of maxillofacial fungal infections in patients with diabetes treated for SARS-CoV-2, with a strong association with corticosteroid administration. Neutropenia, malignancy, organ transplant, immunosuppression, and chemotherapy are all risk factors for opportunistic fungal infection [43]. In contrast; Szaboa et al. [33] reported an increased incidence of pulmonary-associated aspergillosis in elderly men.

As regards the healthcare-related risk factors, our results showed a significant association between fungal coinfection and high doses or long-term use of corticosteroids; prolonged use of multiple antibiotic combinations; use of tocilizumab; the length of stay in the ICU; both invasive and noninvasive mechanical ventilation; and other invasive medical procedures, including central venous line insertion and parenteral nutrition. This is in accordance with what is reported in the literature: fungal infections are correlated with hospital or ICU admission and intubation [43], the use of various respiratory supports, such as invasive or non-invasive ventilation, and the length of ICU stay [47]. Another indirect connection between the concomitant rise in COVID-19 and *mucormycosis* is the proliferation of fungal spores in the water used in oxygen humidifiers. In hospital water, fungi such as Mucorales can grow and reproduce [49].

High doses and long-term use of corticosteroids predispose to bacterial and fungal infections [51]. Tocilizumab, a humanized monoclonal antibody against soluble and membrane IL-6 receptors, used in the treatment of patients with rheumatoid arthritis, is associated with an increased risk of infection, especially of the respiratory system [52]. Kimming et al. [53] reported in their retrospective study that critically ill COVID-19 patients receiving tocilizumab have a higher risk of secondary fungal infections. Prattes et al. [54] demonstrated tocilizumab, not glucocorticoids, as a risk factor for invasive pulmonary aspergillosis in critically ill COVID-19 patients.

Poor diabetes control, prolonged and/or high-dose steroids and the presence of multiple comorbidities were all significant risk factors for the development of a fungal coinfection in critically ill COVID-19 patients, according to the results of a multivariate analysis.

Guaraldi et al. [55] showed that tocilizumab increases the incidence of candidemia and IFIs. In a Spanish cohort, treatment with tocilizumab alone or along with glucocorticoids increased the risk of systemic candidiasis (p-value = 0.05; 0.010, respectively) [56]. In contrast to our finding, Xu et al. [57] traced no secondary fungal infections in a small series of 21 severe and critically ill COVID-19 patients treated with tocilizumab and revealed no significant difference in fungal infections between patients who received tocilizumab or not.

Regarding the outcome, our results showed that the length of ICU stays and mortality rates were significantly higher in COVID-19 patients with fungal coinfection than in the non-fungal group (63.9%) versus 12.9%. Furthermore, the mortality rates varied according to the type of fungal coinfection, with the highest rates in a patient with *mucormycosis* (100%), followed by multisite candida infection and *candidemia* (88.9% and 83.3%), then *aspergillosis* (81.8%), while mortality rates were lower in the patients with pulmonary and/or urinary *candidiasis* without *candidemia* (35%).

The high mortality rates in our study are in line with those reported by Segrelles-Calvo et al. [56], who reported a mortality rate of 86% in patients with CAPA. Another study reported a mortality rate of 66.6% in COVID-19 patients associated with aspergillosis and an 80% mortality rate in patients who developed candidemia [40]. Also, in other reports, COVID-19-associated Candida auris outbreaks have resulted in mortality rates ranging from 30 to 83% in those with candidemia [58, 59].

Regrettably, even with adjunct surgery, the death rate for mucormycosis patients is high in COVID-19 patients, where mortality rates are greater than 80% depending on the location of the disease, such as pulmonary or disseminated mucormycosis [60].

An analysis of the bad outcomes among the present *mucormycosis* cases was similar to an Egyptian study analysis [60] during the third pandemic wave, when the rate of *mucormycosis* linked with COVID-19 was higher (8.25%) with mortality greater than 90%. Multi-organ failure and septicemia accounted for the deaths from *mucormycosis*. A delayed surgical procedure, especially in our critical cases with difficult interventions and expensive extended antifungal therapy, led to a poor outcome.

The appropriate identification of fungal invasiveness using diagnostic bronchoscopy could not be performed in the investigated ICU because of the potential for cross-contamination, which was the main limitation of this study. Serum or bronchial lavage galactomannan (GM) was not available at the time of investigation to help diagnosis. Despite having high negative predictive values (97–99%), the predictive invasiveness clinical scores, used as a tool for starting empirical antifungal therapy at our university, have poor positive predictive values [61, 62].

## Conclusion

Fungal coinfection is a common complication of critically ill COVID-19 patients admitted to the ICU, especially those with poor diabetic control, concomitant use of prolonged and/or high-dose steroids, and multiple associated comorbidities and. Candidiasis, *aspergillosis*, and *mucormycosis* are the most prevalent COVID-19-associated fungal infections and have a great impact on mortality rates. Thus, screening critically ill COVID-19 patients is crucial to avoid delays in diagnosis and treatment that might worsen their prognosis.

## Data Availability

Data can be obtained from the corresponding authors upon reasonable request.

## List of abbreviations

COVID-19: Corona Virus Disease (2019)
ICUs: intensive care units
ZU: Zagazig University
COPD: chronic obstructive pulmonary disease
SARS-CoV-1: severe acute respiratory syndrome coronavirus 1
CKD: chronic kidney disease
ACE2: angiotensin-converting enzyme 2
TMPRSS2: transmembrane protease serine 2
IL: interleukin
CD: cluster of differentiation
RT - PCR: Reverse transcription polymerase chain reaction
CRP: C-reactive protein
BMI: body mass index
SARS-CoV2: Severe Acute Respiratory Syndrome Coronavirus 2
ETA: Endotracheal Aspirate
CDC: Centers for Disease Control and Prevention
KOH: potassium hydroxide
SDA: Sabouraud dextrose agar
LPCB: lactophenol cotton blue
ARDS: acute respiratory distress syndrome
IFIs: invasive fungal infections
DM: diabetes mellitus
CAPA: COVID-19 associated pulmonary aspergillosis
MV: mechanical ventilation.
